# Uterine Hemorrhage Following Abortion

**Published:** 1897-01

**Authors:** J. Hobart Egbert

**Affiliations:** Holyoke, Mass.


### Uterine Hemorrhage Following Abortion.

BY J. HOBART EGBERT, A. M., M. D., PH. D., HOLYOKE, MASS.

How replete with meaning are the following lines, which we translate from the Latin of Virgil: "Happy, indeed, is he who, having determined the causes of things, has put Fate and inexorable Destiny under his feet."

Herein we have expressed, not only the secret of happiness, but also of success, and may, also, trace the essentials of true prophecy, and learn the ideal method for determining the plan of present action, and of foretelling future effects. The days of astrology and chance, in matters pertaining to health, are past, and all blind speculations, and false doctrines, are being cast into the mouldy lumber-room of obsolete superstition by the mighty hand of knowledge. Surgery is an exact science, and medicine is rapidly approaching a similar standard. How readily the proper and best methods of relief suggest themselves to the intelligent surgeon, when once the real condition—the *status præsens*—is appreciated! The unerring finger of knowledge points out the numbers on the teetotum of physical conditions, and in the light of science, we read the numbers indicated, and lo! they yield us the keys, by means of which art may unlock the coffers of health and happiness.

Through the columns of a recent issue of one of our medical journals, a practitioner asks, what should be done for a female patient who miscarried two months ago, and who still suffers from more or less profuse hemorrhage from the uterus, both during and in the intervals of menstruation? Surely, he should be enlightened, for in these days of frequent abortions these cases are numerous.

Another case, to which we would direct attention, was one in the charge of a young physician, to whose aid we were summoned in the wee small hours of the morning, a very short time ago. His patient—a young married lady—had recently aborted, had flowed excessively and continuously for three days, and was now, apparently, *in articulo mortis.* All efforts to check the hemorrhage had been unsuccessful.

We mention these cases, not because they are rare, but rather because they are typical and common, and because they call for positive and definite treatment—thereby affording a suitable text for our remarks. Many practitioners will recall numerous almost identical cases, which they have encountered, and, perhaps, readily relieved; while some may still be in search of further light, and to these we address our words.

Hemorrhage from the uterus following abortion may be due to the presence, within the cavity of the uterus, of fragments of placenta or fetus; to retention of entire placental membranes; or may arise from subinvolution. When, in cases of abortion, the uterus has not been entirely emptied of the developing fetus and its appendages, the retained portions not only prevent retraction of the uterus, and act as veritable drainage tubes for the immediate perpetuation of the hemorrhage, but, by their subsequent disintegration, and the irritation which they occasion, they induce inflammation of the endometrium, *i. e.*, *endometritis simplex*, or *endometritis granulosa.* Endometritis is most likely to result when the retained particles are not sufficiently large to occasion serious primary hemorrhage, and thus lead to their early and complete removal.

The immediate treatment of a given case may vary somewhat with the conditions presented at the time when relief is required, but in every case the treatment must eventually be directed to the removal of the cause of the hemorrhage. If, in cases of recent abortion, digital examination, or the introduction of a sound, reveals the presence of a large piece of placenta—or even entire placenta—within the cavity of the uterus, this must be carefully and thoroughly removed. This may often be effected by means of the index finger, gently inserted through the os, and passed to the point of attachment. The blunt uterine curet and placental forceps are often of assistance in detaching the mass, but the finger is the safest, and should be given preference in most cases. Retained fragments will sometimes be spontaneously ejected upon a well applied tampon, but in cases where profuse hemorrhage has induced a condition bordering on collapse, even the vaginal tampon cannot always be depended upon as a means of checking the hemorrhage. With the cavity of the uterus duly emptied, but little further local treatment—aside from the use of a warm, cleansing, antiseptic vaginal douche—is likely to be demanded; but should the hemorrhage persist, astringent applications, tamponing the vagina and the internal administration of ergot are in order.

In long standing cases of retained debris and chronic granulation of the endometrium, thorough curetting of the cavity of the uterus is the proper method of treatment. Rest in the recumbent posture, the administration of ergot and the local use of tampons and astringents will frequently afford temporory relief, but in almost all cases the hemorrhage recurs at each menstrual period, if not before. In cases of subinvolution the administration of ergot and uterine alteratives, with proper local treatment, and rest in bed, will often effect a cure, but the patient should not be permitted to leave her room until all trace of hemorrhage has disappeared. A favorite prescription in these cases is:

Fluid extract of ergot (Squibb's)..............2 fluidrams.
Fluid extract of viburnum prunifolium......2 fluidounces.
Tincture of cinnamon, enough to make......4 fluidounces.
Mix.

DOSE:—Teaspoonful in hot water from two to six times a day.

Locally, douches of hot water containing antiseptics and mild astringents, and the nightly insertion of an antiseptic vaginal tablet.

To return, ere concluding, to the two cases to which reference has already been made. The first is evidently a subject for the curet—also, perhaps, for general treatment. The second case was one of retained placenta, and the offending mass was removed without delay. Owing to the extreme exhaustion of the patient from loss of blood, stimulants were administered, also ergot in the following combination:

Fluid extract of ergot........ ..................½ fluidounces.
Tincture of digitalis.............................2 fluidrams.
Distillate of witchhazel.........................1 fluidounce.
Tincture of cinnamon, enough to make......4 fluidounces.
Mix.

Dose:—1 fluidram as necessary.

Liquids—hot tea with plenty of milk, beef tea from the extract, expressed beef juice, etc.—were given at short intervals in divided amounts. The patient made a good and uneventful recovery.

Finally, abortion must ever be regarded as a serious condition, worthy most careful consideration. In fact, neglect and improper treatment of this condition are probably the most fruitful sources of chronic uterine diseases and of menstrnal disorders encountered by woman. The treatment of abortion is practically always the same, to-wit: first and foremost, to prevent it when possible, but, when abortion is inevitable, to favor expulsion of the entire fetal mass, remembering that the treatment is never completed until the uterus is both contracted and retracted, and its cavity free from granulations and debris.—*Philadelphia Polyclinic.*

